# Machining Performance of Cryogenic Minimum Quantity Lubrication-Assisted High-Speed Milling 2343ESR Mold Steel

**DOI:** 10.3390/ma19020319

**Published:** 2026-01-13

**Authors:** Ziyi Li, Weimin Dong, Shengwei Ba, Liang Li, Guolong Zhao

**Affiliations:** College of Mechanical and Electrical Engineering, Nanjing University of Aeronautics and Astronautics, Nanjing 210016, China; liziyi@nuaa.edu.cn (Z.L.); dwm5230@nuaa.edu.cn (W.D.); bashengwei@nuaa.edu.cn (S.B.); liliang@nuaa.edu.cn (L.L.)

**Keywords:** 2343ESR mold steel, cryogenic minimum quantity lubrication (CMQL), cutting performance, surface defects, tool life

## Abstract

To improve the machinability of 2343ESR mold steel and promote environmentally sustainable machining, this study systematically investigates its cutting performance in high-speed milling assisted by cryogenic minimum quantity lubrication (CMQL). A series of comparative high-speed milling experiments were conducted under dry cutting and CMQL conditions to elucidate the synergistic cooling and friction-reducing mechanisms of CMQL in the cutting zone. The effects of cutting parameters on key indicators including cutting forces, surface roughness, and tool life were investigated. Tool wear mechanisms were further analyzed and compared based on microscopic observations of workpiece surface damage and tool wear morphologies. The results show that, compared with dry cutting, CMQL reduces resultant cutting force by approximately 15.7–25.2% and surface roughness by about 14.6–29.9%. With the assistance of CMQL, the machined surface defects such as tearing, spalling and microcracks were effectively suppressed. In addition, adhesive wear and flank wear of the tool were significantly retarded, thereby achieving a significant improvement in tool life. These findings demonstrate that CMQL-assisted high-speed milling is a high-efficiency, high-quality and environmentally friendly machining technology with broad application potential for 2343ESR mold steel.

## 1. Introduction

2343ESR mold steel is a high-performance Cr-alloyed hot-work mold steel produced by electroslag remelting (ESR). It exhibits high hardness and good wear resistance. The increased chromium content enhances its oxidation resistance and performance in mildly corrosive environments [1,2]. Meanwhile, the ESR process enhances material cleanliness and microstructural homogeneity, decreases internal defects, and consequently increases fatigue strength and service life [3]. In addition, this steel exhibits good machinability and low distortion after heat treatment, making it suitable for manufacturing complex dies. Owing to these attributes, it is widely used in precision mold applications such as plastic injection molds, die-casting molds and blow molds [4,5], as well as in cutting tools, wear-resistant industrial components and high-stress, corrosion-resistant parts in the automotive and aerospace sectors [6]. However, its high hardness and wear resistance greatly increase the machining difficulty, and its relatively low thermal conductivity promotes heat accumulation in the cutting zone, which can lead to rapid tool wear or thermal damage to the machined surface [7,8]. Therefore, extending tool life and improving surface quality are key issues that must be addressed when machining this steel.

To improve machining efficiency, conventional processes commonly employ large amounts of cutting fluid for cooling, lubrication, and chip evacuation. However, the resulting waste fluid pollution, excessive energy consumption, and health risks have become increasingly serious concerns [9,10,11]. In recent years, various approaches have been proposed to reduce or even eliminate the use of cutting fluids, of which dry cutting and minimum quantity lubrication (MQL) are the most widely adopted strategies [12]. For difficult-to-machine materials, dry cutting often leads to shortened tool life and deterioration of the machined surface [13]. In contrast, MQL can form a thin oil film at the tool-workpiece interface, thereby reducing friction and significantly reducing cutting fluid consumption. Nevertheless, its limited cooling capacity may result in high temperatures in the cutting zone and degradation of the lubricating performance of the oil film at the tool-workpiece interface [14]. Meanwhile, cryogenic cooling can rapidly remove heat from the cutting zone via the latent heat of vaporization of the cryogen, but it tends to induce surface hardening and increase machining difficulty [15,16]. To overcome the individual limitations of these methods, recent studies have proposed the integration of cryogenic cooling with MQL—known as cryogenic minimum quantity lubrication (CMQL)—a technique that exploits their complementary advantages in lubrication and cooling.

A growing body of research has evaluated the potential of CMQL across various materials and processes. Ge et al. [17] investigated the application of CMQL in machining Ti-6Al-4V thin-walled parts. Their experimental results showed that CMQL significantly reduced the cutting load and deformation of the thin walls and improved tool wear resistance and machined surface quality compared with flood cooling and MQL. However, due to the low thermal conductivity and unique mechanical properties of titanium alloys, the applicability of these findings to high-hardness steels remains uncertain. Robson et al. [18] studied helical milling of holes in Inconel 718 using hybrid cryogenic and MQL cooling-lubrication strategies. Among the tested conditions, CMQL provided the longest tool life and the best surface quality, but at the expense of higher cutting forces, which were attributed to embrittlement of the material at cryogenic temperatures. This suggests that the effectiveness of CMQL is material-dependent and can influence tool–workpiece interactions in complex ways. Alborz et al. [19] developed a novel hybrid cryogenic MQL cooling-lubrication technique and proposed a new tool life model based on tool wear. Compared with state-of-the-art flood cooling, the tool life increased by a factor of 30 and productivity was improved by 50% as well. Nicolai et al. [20,21] conducted a comprehensive evaluation of tool wear, cutting torque, and surface integrity when machining Ti-6Al-4V using CMQL, MQL, and flood cooling. Their results indicated that, when the cryogenic medium and the MQL oil-mist composition were identical, CMQL offered clear advantages over both flood cooling and MQL. Nimel et al. [22] reported, based on systematic experiments, that CMQL was the most effective cooling strategy, as it minimized friction and produced the best surface finish. Under CMQL, flank wear was reduced by 51–55%, 37–47% and 26–33% compared with dry cutting, MQL, and CO_2_ cooling, respectively. Çağrı et al. [23] showed that in turning alloy 625, CMQL reduced surface roughness by 24.82% relative to cryogenic cooling, while tool wear under MQL and CMQL decreased by 50.67% and 79.60%, respectively. However, MQL was more effective than cryogenic cooling in reducing cutting tool wear. Zhang et al. [24] investigated tool wear and white layer formation during hard milling of AISI H13 steel under dry and CMQL conditions. CMQL largely eliminated white layer formation, a severe subsurface damage mechanism caused by rapid thermal cycling. However, the research is limited to low-speed milling (130 m/min), and no systematic investigations have been reported on cutting force components, surface morphology defects, or high-speed performance related to industrial mold production.

Considering the industrial importance and machining difficulty of 2343ESR mold steel, as well as the limitations of conventional flood cooling, dry cutting, and MQL in machining hot-work mold steel, the development of sustainable alternatives such as CMQL is urgently needed. Despite promising results in other materials, the lack of systematic studies on CMQL’s performance in high-speed milling of hot-work steels highlights a significant knowledge gap. This study provides a systematic investigation of CMQL behavior and mechanisms in high-speed milling of 2343ESR mold steel, emphasizing its influence on cutting forces, surface roughness, and surface integrity. The effects of cutting speed and feed rate on the three components of cutting force are examined by comparing experimental results obtained under dry cutting and CMQL conditions. Combined with surface roughness measurements and microstructural observations of the machined surface, the advantages of CMQL in improving surface integrity are demonstrated, and its effects on tool wear behavior and tool life extension are further explored. By analyzing the interrelationships among cutting parameters, surface quality, and tool life, this study provides a basis and technical reference for high-efficiency, high-precision, and environmentally sustainable machining of 2343ESR mold steel.

## 2. Materials and Methods

This section describes the experimental setup and parameters. High-speed milling tests were carried out under two machining environments, dry cutting and CMQL, to investigate the effects of CMQL on cutting forces, surface roughness, and surface quality. By comparing tool life under different cutting environments, this study evaluates the advantages of cryogenic minimum quantity lubrication for high-efficiency, high-quality, and environmentally sustainable machining.

### 2.1. Workpiece and Cutting Tool

The workpiece material used in this study was 2343ESR high-strength hot-work mold steel (Changzhou Xingyu Automotive Lighting System Co., Ltd., Changzhou, China) with a hardness of approximately 48 HRC. Its chemical composition is listed in Table 1. Block specimens with dimensions of 80 mm × 60 mm × 60 mm were prepared, and their surfaces were ground prior to milling to minimize the influence of the initial surface condition on the experimental results.

The milling experiments were performed using a three-flute, 8 mm diameter TiAlN-coated carbide end mill for high-speed machining of hardened steels. The TiAlN coating, with a thickness of about 3 μm, offered excellent wear and oxidation resistance. End milling was employed to enable a direct comparison of cutting performance under dry cutting and CMQL using identical tool parameters. Table 2 lists the geometrical parameters of the cutting tool used in this study.

### 2.2. Experimental Design

**Figure 1:**
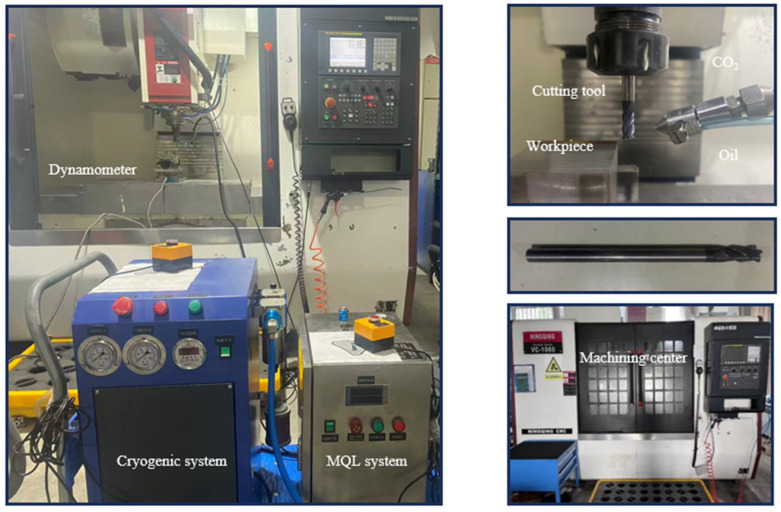
Experimental setup and cryogenic system.

End milling experiments under dry cutting and CMQL conditions were carried out on a Ningqing VC1060 vertical machining center (Ningqing Aerospace Intelligent Equipment Co., Ltd., Nanjing, China). Cutting forces (F_x_, F_y_, F_z_) during milling were measured with a KISTLER 9443B piezoelectric three-component dynamometer (Kistler Instrumente AG, Winterthur, Switzerland). Each cutting condition was repeated three times to ensure statistical reliability with mean values and standard deviations calculated from the steady-state portions of the force signals. The surface roughness of the machined surfaces was measured using a portable TR200 profilometer (Time Group Inc., Beijing, China). For each test, the arithmetic mean roughness (Ra) was recorded at three different positions along the feed direction, and the mean value was reported. The surface morphology after milling under different cutting environments was examined using a scanning electron microscope (SEM, Regulus 8220, Hitachi High-Technologies Corp., Tokyo, Japan). During the tool wear tests, flank wear was measured using an optical microscope (OM, Shenzhen Sanqiang Taida Optical Instrument Co., Ltd., Shenzhen, China). The experimental setup and the cryogenic minimum quantity lubrication system used in this study are shown in Figure 1.

### 2.3. Experimental Parameters

This study evaluated the machinability of 2343ESR mold steel through a comparison of high-speed end milling under different cooling and lubrication conditions. A full-factorial design with 16 experiments was adopted to examine two typical machining environments—dry cutting and CMQL. For each cutting condition, the three components of the cutting force were recorded, the surface roughness was measured, and the machined surfaces were examined microscopically to analyze changes in surface integrity and the mechanisms of defect formation. In addition, tool life tests were conducted under identical cutting parameters to compare tool wear mechanisms between different machining environments. These investigations provide a technical basis for applying CMQL to high-speed milling of high-hardness mold steels. The cutting parameters used in the experiments are listed in Table 3. The cutting speeds (225–300 m/min) and feed rates per tooth (0.005–0.02 mm/z) were selected based on the ASSAB tool guide and prior CMQL studies on high-hardness steels, covering the recommended high-speed milling range for mold steels while balancing production efficiency and tool life, and ensuring representative experimental conditions without excessive vibration.

## 3. Results

### 3.1. Cutting Force

**Figure 2:**
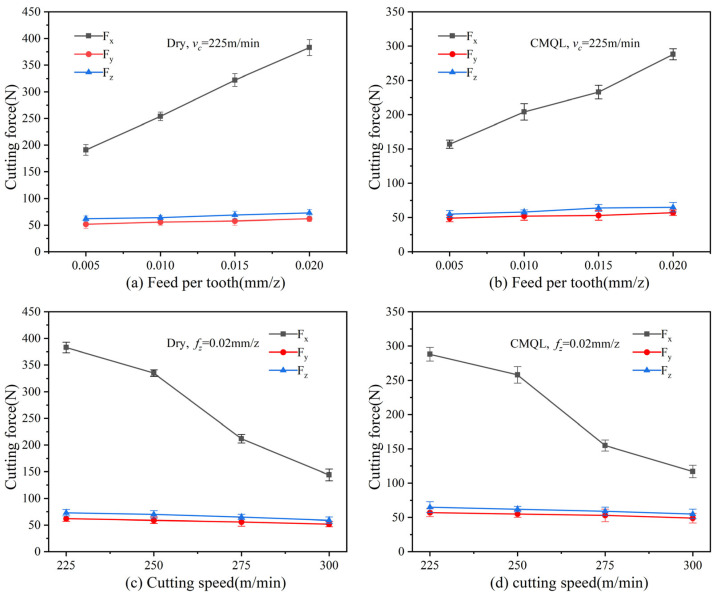
Cutting force obtained under various cutting parameters and cooling lubrication methods.

The influence of cutting parameters on the cutting forces under different cutting environments is shown in Figure 2. A comparison of the cutting-force and cutting-speed curves for dry cutting and CMQL milling shows that cutting forces decrease overall with increasing cutting speed in both cases. As the cutting speed increases, the elevated temperatures in the cutting zone, as reported in prior studies [14,15,16,25], promote thermal softening of the workpiece material and a reduction in shear stress, which facilitates material removal and thus reduces the cutting forces. By contrast, the cutting forces increase with increasing feed per tooth. A higher feed per tooth shortens the time available for heat dissipation in the cutting zone and increases the energy required to remove a larger volume of material in a shorter time, which in turn results in higher cutting forces.

Under CMQL conditions, the cutting forces are significantly lower than those under dry cutting for the same cutting parameters. The measurements show that, compared with dry cutting, CMQL reduces the main cutting force F_x_ by approximately 17.8–27.6%, the radial force F_y_ by 5.4–8.6% and the axial force F_z_ by 6.8–11.4%, with the corresponding resultant cutting force decreasing by 15.7–25.2% under identical cutting parameters. In dry cutting, the absence of cooling and lubrication significantly increases friction between the rake face and the chip, as well as between the flank face and the machined surface. Elevated cutting temperatures promote frequent adhesion and local welding, thereby substantially increasing cutting resistance. In contrast, under CMQL the cryogenic gas stream rapidly removes heat from the cutting zone (as reported in [25]), while the small amount of lubricant, driven by the high-speed gas flow, penetrates into the tool–chip contact zone and forms a stable oil film. According to the capillary action mechanism, the MQL lubricant can form a continuous lubricating film that reduces friction between the chip and the rake face, stabilizes the cutting process and consequently leads to a significant reduction in cutting forces.

### 3.2. Surface Roughness

**Figure 3:**
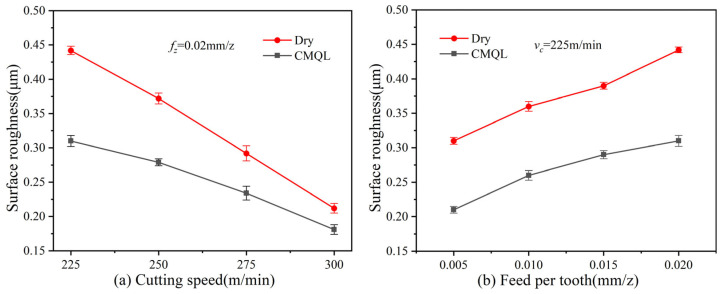
Surface roughness obtained various cutting parameters and cooling lubrication methods.

Figure 3 illustrates the surface roughness (Ra) measured during high-speed milling of 2343ESR mold steel under different cutting parameters and cutting environments. As the cutting speed increases, the contact time between the cutting edge and the workpiece decreases, which hinders the stable formation of a built-up edge and reduces friction and adhesion at the interfaces. Consequently, the surface roughness decreases with increasing cutting speed. In contrast, increasing the feed per tooth increases the material removal rate and raises cutting resistance, resulting in higher surface roughness.

Compared with dry cutting, CMQL results in lower surface roughness under the same cutting parameters. The Ra values under CMQL are approximately 14.6–29.9% lower than those under dry cutting. It is observed that, in CMQL machining, when the cutting speed increases from 225 to 300 m/min, the reduction in surface roughness compared with dry cutting decreases from about 29.9% to 14.6%. This can be attributed to the fact that, at high cutting speeds, the residence time of the coolant–lubricant at the tool–chip interface is shortened, making it difficult to maintain a stable boundary oil film. In addition, chatter induced by high cutting speeds also affects the surface topography and partially offsets the improvement provided by CMQL, resulting in a markedly smaller reduction in roughness at 300 m/min.

**Figure 4:**
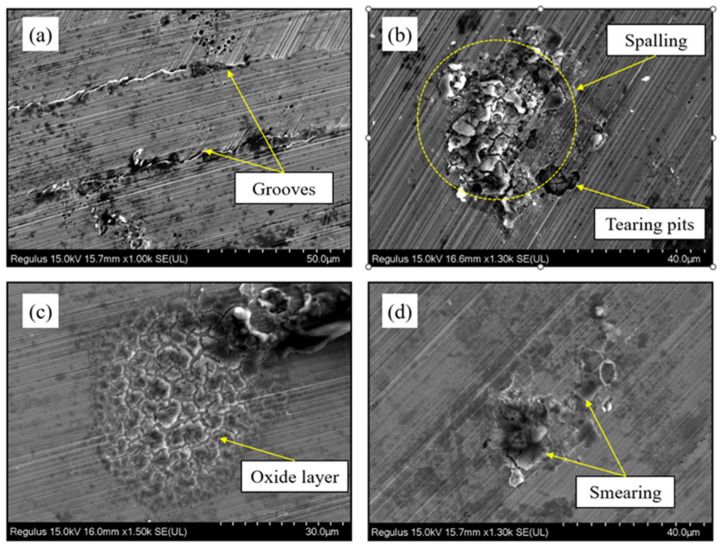
Surface defects of 2343 ESR mold steel under dry cutting conditions. (**a**) Plowing grooves aligned with cutting direction; (**b**) Tear-out pits and local spalling caused by strong adhesion at the tool–chip interface; (**c**) Oxide film formed by high-temperature oxidation and friction, showing cracking upon cooling; (**d**) Local smearing and pile-up of substrate material caused by plastic flow and accumulation.

Under dry cutting conditions, the machined surface exhibits several typical defects, including plowing grooves, tear-out pits and local spalling. The deeper grooves are mostly aligned with the cutting direction and are attributed to plowing and extrusion of the surface material by the cutting edge under high stress and elevated temperature (Figure 4a). Tear-out pits and spalled regions are associated with strong adhesion at the tool-chip interface during cutting. Under high cutting forces, the adhered layer is pulled off, accompanied by severe local plastic deformation and fracture of the surface material (Figure 4b). The formation of an oxide film is mainly attributed to the elevated temperature in the cutting zone: under high-temperature oxidation and friction, the surface metal reacts with oxygen in the environment to form a dense or discontinuous oxide scale, which subsequently cracks upon cooling (Figure 4c). In addition, local smearing and pile-up of the substrate material are observed on the surface (Figure 4d), indicating that the surface layer undergoes plastic flow under high stress. The softened material is drawn by the cutting edge or the chip and smeared onto neighboring regions or extruded and accumulated along groove edges and around micro-pits, thereby forming characteristic smearing and pile-up features. The superposition of these defect types gives rise to relatively poor surface integrity of 2343ESR mold steel under dry cutting conditions. From an application perspective, plowing grooves and tear-out pits initiate fatigue cracks under cyclic loading, reducing precision mold service life. Oxide films compromise corrosion resistance and promote mold sticking. Smearing and pile-up cause dimensional inaccuracies. These defects degrade mold performance, necessitating costly post-processing (e.g., polishing, re-machining), and limit the applicability of dry cutting in precision mold manufacturing.

**Figure 5:**
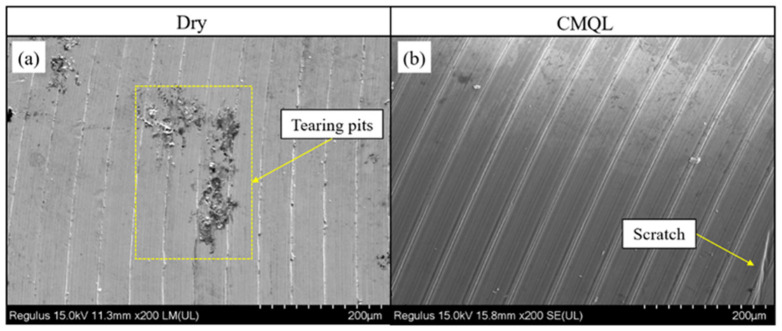
Surface morphologies under (**a**) Dry cutting and (**b**) CMQL conditions (v_c_ = 225 m/min, f_z_ = 0.02 mm/z).

Figure 5 presents SEM images of the machined surfaces under dry cutting and CMQL conditions (v_c_ = 225 m/min, f_z_ = 0.02 mm/z). Under dry cutting, as shown in Figure 5a, the machined surface of the mold steel is dominated by deep plowing grooves and tear-out pits, while local regions exhibit surface spalling induced by high-temperature adhesion and severe plastic deformation. In contrast, under CMQL the cooling medium in the form of compressed gas carries an oil mist into the cutting zone and its vicinity. As a result, the cooling and lubrication conditions are markedly improved, and the cutting temperature and adhesive wear are effectively suppressed (Section 3.1). Only a few fine adhered particles and shallow micro-pits are observed locally. No obvious tear-out pits or spalling can be seen, indicating a pronounced improvement in surface integrity.

### 3.3. Tool Life

**Figure 6:**
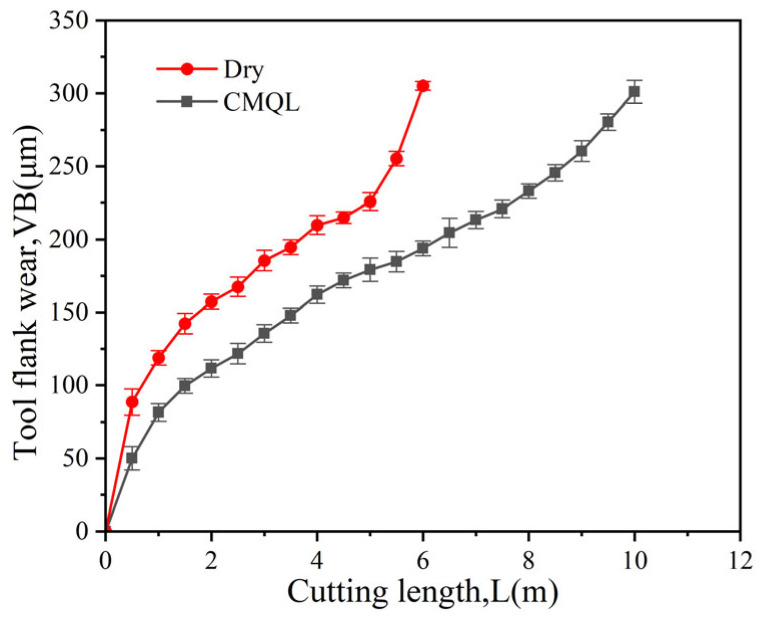
Evolution of tool flank wear width with cutting length.

In the tool life tests, identical cutting parameters (v_c_ = 225 m/min, f_z_ = 0.02 mm/z, ap = 0.5 mm, ae = 4 mm) were employed under dry cutting and CMQL to directly compare the influence of CMQL on tool wear. After each pass using the same cutting length, the tool tip was cleaned with anhydrous ethanol to remove adhered chips and other debris contaminants. Because tool wear enters a severe wear stage and the surface quality deteriorates once the wear reaches a certain level, an average flank wear land width of 0.3 mm was adopted as the tool life criterion to ensure an acceptable surface finish. The evolution of flank wear under different machining environments is shown in Figure 6. Under CMQL, the tool life increased from about 6 m to approximately 10 m of cutting length (roughly 1.7 times that under dry cutting), and the steady-wear stage exhibited the longest duration.

**Figure 7:**
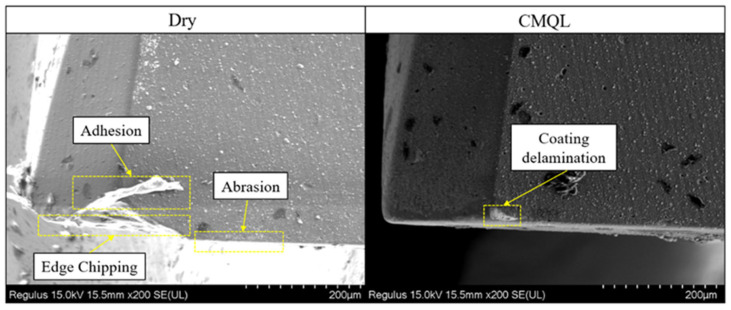
Wear morphology of tool flank face under dry and CMQL conditions.

Figure 7 shows SEM images of flank wear on the TiAlN-coated tools under dry cutting and CMQL conditions. In high-speed milling, the elevated shear stress on the tool promotes adhesive wear and edge chipping, which become the dominant wear modes. Under dry cutting, a wide flank wear land accompanied by pronounced edge chipping was observed. The region near the cutting edge is highly uneven, with grooves oriented along the cutting direction and local coating spalling, indicating that abrasive wear and adhesion-induced spalling dominate under high cutting temperature and contact stress. In contrast, under CMQL the flank wear land is markedly narrower, the surface profile is relatively smooth, and the overall wear level is much lower than that under dry cutting. This demonstrates that CMQL effectively mitigates abrasive and adhesive wear on the flank face by reducing cutting temperature and interfacial friction, thereby significantly decreasing tool wear.

## 4. Conclusions

(1) CMQL high-speed milling markedly reduced the cutting loads when machining 2343ESR mold steel. Compared with dry cutting, all three orthogonal force components decreased: the main cutting force F_x_ decreased by approximately 17.8–27.6%, the radial force F_y_ by 5.4–8.6% and the axial force F_z_ by 6.8–11.4%, the corresponding resultant cutting force decreased by 15.7–25.2%. CMQL weakens adhesion and friction at the tool–chip interface and improves chip evacuation, thereby effectively reducing the mechanical loading on the tool.

(2) Under identical cutting parameters, the surface roughness obtained under CMQL was significantly lower than that under dry cutting, with Ra reduced by about 14.6–29.9%. The surface topography changed from deep plowing grooves, tear-out pits and local spalling under dry cutting to predominantly shallow abrasive scratches and slight smearing of the substrate under CMQL. Both the number and size of micro-defects decreased markedly, and surface integrity was greatly improved. This indicates that CMQL can effectively suppress surface damage and is beneficial for improving the surface quality.

(3) Optimal performance occurs at the cutting speed of 225 m/min and a feed rates per tooth of 0.02 mm/z (maximum 29.9% Ra reduction and maximum 25.2% reduction in resultant cutting force), with diminishing returns at 300 m/min due to shortened lubricant residence time. Provides guidance for the actual parameter selection of 2343ESR milling.

(4) In terms of tool performance, CMQL increased the tool life from about 6 m to approximately 10 m of cutting length, roughly 1.7 times that under dry cutting, and effectively suppressed flank wear, edge chipping, and coating spalling on the TiAlN-coated tools, thereby improving tool utilization efficiency and reducing machining costs.

This study indicates that CMQL is a promising green machining strategy for high-speed milling of hot-work mold steels, enabling lower cutting loads, better surface integrity and longer tool life than dry cutting, and thus offering clear benefits for high-efficiency and sustainable manufacturing.

## Figures and Tables

**Table 1:** Chemical composition of 2343ESR mold steel.

Element	C	Mn	Si	Cr	Mo	V	P	S	Fe
wt.%	0.41	0.49	0.85	5.47	1.11	0.6	0.01	0.01	Bal.

**Table 2:** Geometrical parameters of the cutting tool.

Parameter	Value
Tool diameter	8 mm
Number of teeth	3
Length of coating	20 mm
Nose radius	0.5 mm
Rake angle	12°
Clearance angle	10°
Helix angle	60°

**Table 3:** Experimental conditions and process parameters.

Parameters	Conditions
Work Material	2343ESR mold steel
Cutting tool	Solid carbide endmill with single-layer TiAlN
Cutting speed (v_c_)	225, 250, 275, 300 (m/min)
Feed per tooth (f_z_)	0.005, 0.01, 0.015, 0.02 (mm/z)
Depth of Cut (ap)	0.5 mm
Cooling environment	Dry, CMQL
CO2 Pressure	0.6 MPa
MQL oil flow rate (Q)	60 mL/h
MQL oil	Blaser Vascomill MMS FA 1

## Data Availability

The original contributions presented in this study are included in the article. Further inquiries can be directed to the corresponding author.

## References

[1] Hamdi A., Yapan Y.F., Uysal A., Merghache S.M. (2023). Machinability assessment and optimization of turning AISI H11 steel under various minimum quantity lubrication (MQL) conditions using nanofluids. Int. J. Adv. Manuf. Technol..

[2] Balasuadhakar A., Kumaran S.T., Ali S. (2025). Sustainable Cooling Strategies in End Milling of AISI H11 Steel Based on ANFIS Model. Machines.

[3] Yang W., Liu Z., Li Y., Sun Y., Wang F., Li B. (2026). Effects of fill ratio on magnetohydrodynamics and macrosegregation behavior in electroslag remelting. Int. J. Therm. Sci..

[4] Huber F., Bischof C., Hentschel O., Heberle J., Zettl J., Nagulin K.Y., Schmidt M. (2019). Laser beam melting and heat-treatment of 1.2343 (AISI H11) tool steel-microstructure and mechanical properties. Mater. Sci. Eng. A.

[5] Balaško T., Vončina M., Burja J., Batič B.Š., Medved J. (2021). High-temperature oxidation behaviour of AISI H11 tool steel. Metals.

[6] Platt T., Biermann D. (2025). Model-based optimization of micromilling AISI H11 tool steel: A comprehensive study of wear and its impact on surface quality. Wear.

[7] Zhao G., Zhao B., Ding W., Xin L., Nian Z., Peng J., He N., Xu J. (2024). Nontraditional energy-assisted mechanical machining of difficult-to-cut materials and components in aerospace community: A comparative analysis. Int. J. Extrem. Manuf..

[8] Platt T., Meijer A.L., Biermann D. (2024). Experimental Investigation on the Surface Integrity in Micromilling AISI H11 Tool Steel. Procedia CIRP.

[9] Xin L., Zhao G., Nian Z., Yang H., Li L., He N. (2025). Physics-based modeling and mechanism of polycrystalline diamond tool wear in milling of 70 vol% Si/Al composite. Int. J. Extrem. Manuf..

[10] Liu T., Zhao G., Khan A.M., Li L. (2025). Experimental study of machinability of CHSF/PR composites by end milling. Mater. Manuf. Process..

[11] Shokrani A., Arrazola P.J., Biermann D., Mativenga P., Jawahir I.S. (2024). Sustainable machining: Recent technological advances. CIRP Ann..

[12] Duman E., Yapan Y.F., Salvi H., Sofuoğlu M.A., Khanna N., Uysal A. (2024). Investigation of ultrasonic vibration assisted orthogonal turning under dry and minimum quantity lubrication conditions and performing sustainability analyses. J. Clean. Prod..

[13] Zhang J., Zheng Z., Huang K., Lin C., Huang W., Chen X., Xiao J., Xu J. (2024). Field-assisted machining of difficult-to-machine materials. Int. J. Extrem. Manuf..

[14] Ali S.H., Yao Y., Wu B., Zhao B., Ding W., Jamil M., Khan A., Baig A., Liu Q., Xu D. (2025). Recent developments in MQL machining of aeronautical materials: A comparative review. Chin. J. Aeronaut..

[15] Xu K., Yang Y., Feng W., Wan M., Zhang W. (2024). Internal cooling techniques in cutting process: A review. J. Adv. Manuf. Sci. Technol..

[16] Korkmaz M.E., Gupta M.K., Ross N.S., Sivalingam V. (2023). Implementation of green cooling/lubrication strategies in metal cutting industries: A state of the art towards sustainable future and challenges. Sustain. Mater. Technol..

[17] Wu G., Li G., Pan W., Raja I., Wang X., Ding S. (2022). Experimental investigation of eco-friendly cryogenic minimum quantity lubrication (CMQL) strategy in machining of Ti-6Al-4V thin-wall part. J. Clean. Prod..

[18] Pereira R.B.D., Gómez-Escudero G., Calleja-Ochoa A., Pereira O., González-Barrio H., Brandão L.C., de Lacalle L.N.L. (2025). Hybrid cryogenic/MQL helical milling for hole-making of Inconel 718. Results Eng..

[19] Shokrani A., Al-Samarrai I., Newman S.T. (2019). Hybrid cryogenic MQL for improving tool life in machining of Ti-6Al-4V titanium alloy. J. Manuf. Process..

[20] Ostrowicki N., Kaim A., Gross D., Hanenkamp N. (2021). Effect of various cooling lubricant strategies on turning Inconel 718 with different cutting materials. Procedia CIRP.

[21] Gross D., Bigelmaier M., Meier T., Amon S., Ostrowicki N., Hanenkamp N. (2019). Investigation of the influence of lubricating oils on the turning of metallic materials with cryogenic minimum quantity lubrication. Procedia CIRP.

[22] Ross N.S., Ganesh M., Srinivasan D., Gupta M.K., Korkmaz M.E., Krolczyk J.B. (2022). Role of sustainable cooling/lubrication conditions in improving the tribological and machining characteristics of Monel-400 alloy. Tribol. Int..

[23] Yıldırım Ç.V., Kıvak T., Sarıkaya M., Şirin Ş. (2020). Evaluation of tool wear, surface roughness/topography and chip morphology when machining of Ni-based alloy 625 under MQL, cryogenic cooling and CryoMQL. J. Mater. Res. Technol..

[24] Zhang S., Li J., Lv H. (2014). Tool Wear and Formation Mechanism of White Layer When Hard Milling H13 Steel under Different Cooling/Lubrication Conditions. Adv. Mech. Eng..

[25] Liu M., Li C., Zhang Y., An Q., Yang M., Gao T., Mao C., Liu B., Cao H., Xu X. (2021). Cryogenic minimum quantity lubrication machining: From mechanism to application. Front. Mech. Eng..

